# Association between dietary inflammatory index score and cardiovascular-kidney-metabolic syndrome: a cross-sectional study based on NHANES

**DOI:** 10.3389/fnut.2025.1557491

**Published:** 2025-05-09

**Authors:** Huang Yu, Yankun Liu, Tingyi Zhang, Ziyi Guan, Ping Li

**Affiliations:** Department of Cardiovascular Medicine, The Second Affiliated Hospital, Jiangxi Medical College, Nanchang University, Nanchang, Jiangxi, China

**Keywords:** cardiovascular-kidney-metabolic (CKM) syndrome, dietary inflammatory index (DII), NHANES (National Health and Nutrition Examination Survey), cross-sectional study, dietary patterns

## Abstract

**Background:**

Cardiovascular-kidney-metabolic (CKM) syndrome affects 25% of US adults, with chronic inflammation as a key pathophysiological mechanism. While the inflammatory basis of CKM syndrome is established, associations of the energy-adjusted dietary inflammatory index (E-DII) with CKM syndrome remain unexplored in the general population.

**Methods:**

Using data from 7,110 participants in the National Health and Nutrition Examination Survey (2007–2018), we examined the association between E-DII (calculated from dietary recall data) and CKM syndrome (defined as co-occurrence of cardiometabolic syndrome and chronic kidney disease). Multiple logistic regression, restricted cubic spline analyses, weighted quantile sum regression, and quantile g-computation were performed to assess associations and dietary component contributions.

**Results:**

Higher E-DII scores correlated with increased CKM syndrome prevalence (OR: 1.22, 95% CI: 1.09–1.37). The relationship exhibited linearity (*p* for nonlinearity = 0.464). Stratified analyses across demographic and socioeconomic subgroups revealed consistent associations. Component analyses identified alcohol as the dietary factor with the strongest association with CKM syndrome.

**Conclusion:**

The findings demonstrate a significant association between dietary inflammatory potential and CKM syndrome, with alcohol consumption emerging as a key modifiable factor. These results provide evidence-based insights for developing targeted dietary interventions in CKM syndrome prevention.

## 1 Introduction

Cardiovascular-kidney-metabolic (CKM) syndrome, characterized by the complex interplay of metabolic risk factors, chronic kidney disease (CKD), and cardiovascular disease (CVD), represents a critical global health challenge (1). This multifaceted condition affects approximately 25% of the adult population in the United States and imposes a substantial burden on healthcare systems (2). The syndrome progresses through five distinct stages (0–4), with advanced stages (3, 4) significantly associated with elevated risks of all-cause mortality (hazard ratio [HR]: 1.84, 95% confidence interval [CI]: 1.65–2.05) and cardiovascular mortality (HR: 2.50, 95% CI: 2.00–3.12) (3).

Chronic inflammation has been established as a fundamental pathophysiological mechanism underlying CKM syndrome (4). The pathogenesis typically initiates with excessive adipose tissue accumulation, which secretes pro-inflammatory cytokines and promotes oxidative stress, ultimately leading to systemic inflammation (5). This inflammatory state subsequently induces insulin resistance, endothelial dysfunction, and tissue damage, establishing a self-perpetuating cycle that accelerates disease progression across cardiovascular, renal, and metabolic systems (6). Given this inflammatory foundation, identifying modifiable factors that influence systemic inflammation is crucial for effective disease management (7).

Among modifiable risk factors, dietary patterns have emerged as a primary therapeutic target (8). The Dietary Inflammatory Index (DII) represents a validated tool, developed through comprehensive systematic literature review, to quantify the inflammatory potential of individual dietary patterns (9). This comprehensive index incorporates up to 45 dietary parameters, encompassing both pro- and anti-inflammatory components (10), and has demonstrated significant correlations with established inflammatory biomarkers, including C-reactive protein (CRP) and Interleukin-6 (IL-6) (11, 12). To enhance comparability across populations with varying total energy intakes, an energy-adjusted version (E-DII) was developed by standardizing dietary components to 1,000 kilocalories of food consumed. This adjustment addresses the limitation of absolute nutrient intake in the original DII and provides a more precise measure of diet’s inflammatory potential (9). The value of E-DII has been extensively validated through epidemiological studies. Cross-sectional analyses have revealed that individuals with higher E-DII scores had a 29% increased risk of chronic kidney disease (13). Among CKD patients, higher E-DII scores were associated with elevated risks of 5-year all-cause mortality (33%) and cardiovascular mortality (54%) (14). Furthermore, a prospective cohort study of 10,138 participants with 5-year follow-up revealed that higher E-DII scores were significantly associated with a 29% increased risk of metabolic syndrome development and a 55% increased risk of the prevalence of metabolic syndrome (15). In the Fasa Cohort Study (*n* = 10,138), higher E-DII scores were independently associated with a 24% increased likelihood of high cardiovascular risk based on the Framingham Risk Score (16).

Accumulating evidence has established robust associations between DII scores and individual components of CKM syndrome. Meta-analyses have demonstrated significant associations between elevated DII scores and increased risks of CVD, CVD mortality, and all-cause mortality (17). Similarly, higher DII scores were associated with increased risk of developing CKD (18) and were independently linked to increased odds of CKD stages 3–5 (19). Regarding metabolic outcomes, pro-inflammatory dietary patterns were significantly associated with increased risks of metabolic syndrome (MetS), abdominal obesity, hypertension, hyperglycemia, and hypertriglyceridemia (20). Despite these well-documented associations with individual components, the relationship between DII and CKM syndrome as an integrated pathophysiological entity remains largely unexplored.

In this study, utilizing data from the National Health and Nutrition Examination Survey (NHANES), we aimed to: (1) investigate the cross-sectional association between E-DII scores and CKM syndrome among US adults; and (2) evaluate the relative contributions of individual DII components to this relationship. Based on established links between inflammation and CKM pathophysiology, we hypothesized that higher DII scores would be positively associated with CKM syndrome prevalence. This investigation may provide crucial insights for developing targeted dietary interventions in CKM syndrome prevention and management.

## 2 Methods

### 2.1 Research design and population

Data were obtained from the NHANES, a population-based surveillance system conducted by the Centers for Disease Control and Prevention (CDC). This study employed a cross-sectional design, which allows for examination of associations but cannot establish causality between dietary inflammatory potential and CKM syndrome. The study utilized NHANES’ multi-stage probability sampling design to ensure nationally representative data from non-institutionalized US civilians. All participants provided written informed consent, and the study protocol was approved by the National Center for Health Statistics Ethics Review Board.

**Figure 1:**
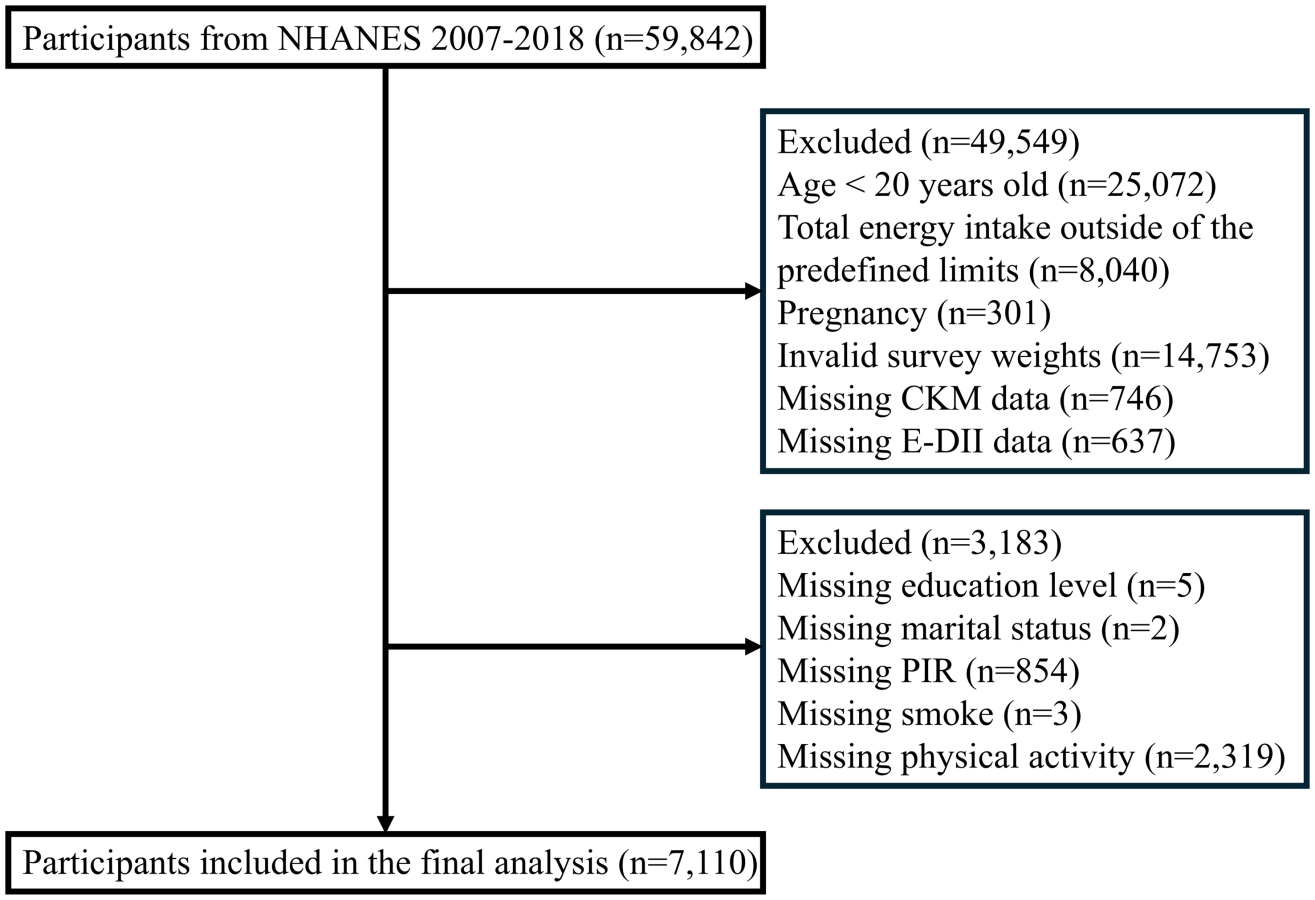
Flow chart of participant’s selection.

The study sample was drawn from six consecutive NHANES cycles (2007–2018), comprising 59,842 initial participants. Sequential exclusions were applied for: age < 20 years (*n* = 25,072); implausible energy intake (men: <800 or >4,200 kcal/day; women: <600 or >3,500 kcal/day (21); *n* = 8,040); pregnancy (*n* = 301); invalid survey weights (*n* = 14,753); missing CKM syndrome data (*n* = 746); incomplete DII information (*n* = 637); and missing covariates (*n* = 3,183). The final analytical sample included 7,110 participants (Figure 1).

### 2.2 Dietary inflammatory index

Dietary assessment was conducted using a two-phase 24-h recall methodology: an initial in-person interview at mobile examination centers, followed by a telephone interview conducted 3–10 days subsequently. The interviews were administered by trained personnel following standardized protocols under the supervision of the NHANES Nutrition Methodology Working Group (22).

The DII was computed using 28 nutritional components: alcohol consumption, total energy intake, carbohydrates, protein, total fat, saturated fatty acids (SFA), monounsaturated fatty acids (MUFA), polyunsaturated fatty acids (PUFA), omega-3 fatty acids, omega-6 fatty acids, cholesterol, dietary fiber, vitamin A, β-carotene, thiamine (vitamin B1), riboflavin (vitamin B2), niacin (vitamin B3), vitamin B6, vitamin B12, vitamin C, vitamin D, vitamin E, folic acid, iron, magnesium, selenium, zinc, and caffeine. These 28 components were selected based on: (1) established biological mechanisms linking them to inflammatory pathways, (2) their significant associations with inflammatory biomarkers in previous research, and (3) their consistent availability in the NHANES dietary data. Of the 45 DII parameters, 17 components were unavailable in the NHANES dataset due to either lack of collection or inconsistent data across survey years. These unavailable components included trans-fat, anthocyanidins, flavan-3-ol, flavones, flavonols, flavanones, isoflavones, eugenol, garlic, ginger, onion, saffron, turmeric, pepper, thyme/oregano, rosemary, green/black tea. Previous studies have demonstrated that the predictive capability of the DII remains robust when using at least 28 parameters, showing comparable performance to analyses utilizing the complete set of 45 components (23). The methodology for DII development and validation has been documented in previous studies (9, 23, 24).

The inflammatory effect scores for the 28 components were derived from an extensive literature review of 1,943 peer-reviewed studies examining their associations with six inflammatory markers (IL-1β, IL-4, IL-6, IL-10, TNF-α, and CRP). The scoring process weighted evidence based on the strength and consistency of these associations. Negative scores indicated anti-inflammatory properties, while positive scores represented pro-inflammatory effects. These scores and their relative weights for the 28 nutritional components used in this analysis are: alcohol (−0.278), total energy intake (0.180), carbohydrates (0.097), protein (0.021), total fat (0.298), saturated fatty acids (0.373), monounsaturated fatty acids (−0.009), polyunsaturated fatty acids (−0.337), omega-3 fatty acids (−0.436), omega-6 fatty acids (−0.159), cholesterol (0.110), dietary fiber (−0.663), vitamin A (−0.401), β-carotene (−0.584), thiamine (−0.098), riboflavin (−0.068), niacin (−0.246), vitamin B6 (−0.365), vitamin B12 (0.106), vitamin C (−0.424), vitamin D (−0.446), vitamin E (−0.419), folic acid (−0.190), iron (0.032), magnesium (−0.484), selenium (−0.191), zinc (−0.313), and caffeine (−0.110).

In accordance with Shivappa et al.’s (9) methodology, these inflammatory effect scores were applied to individual dietary intake data as follows: first, each participant’s dietary intake was standardized against a global reference database to calculate a z-score, which was then converted to a percentile score. Percentile scores were subsequently centered to a mean of zero, multiplied by the corresponding inflammatory effect scores of each dietary component, and summed to derive the overall DII score. To account for measurement bias due to varying energy intakes, the DII was normalized per 1,000 kilocalories to generate E-DII. The final E-DII score is an indicator of the inflammatory potential of the diet, where higher scores reflect more pro-inflammatory dietary patterns. In this study population, the E-DII scores ranged from −2.69 to 6.96.

### 2.3 Diagnosis of cardiovascular-kidney-metabolic syndrome

CKM syndrome currently lacks standardized diagnostic criteria. Its diagnosis requires the concurrent presence of cardiometabolic syndrome (CMS) and CKD in this study (1). This definition aligns with the emerging understanding of CKM syndrome as representing the overlap between cardiovascular, renal, and metabolic pathologies and has been employed in previous NHANES analyses (25).

CMS was defined according to National Cholesterol Education Program Adult Treatment Panel III (NCEP-ATP III) criteria (26), which are widely accepted in epidemiological research, requiring ≥3 of the following: central obesity (objectively measured waist circumference: men ≥102 cm, women ≥88 cm), hypertriglyceridemia (objectively measured triglycerides ≥150 mg/dL), low high-density lipoprotein (objectively measured HDL cholesterol: men <40 mg/dL, women <50 mg/dL), hypertension (objectively measured systolic/diastolic blood pressure ≥130/85 mmHg, self-reported antihypertensive use, or self-reported physician-diagnosed hypertension), and hyperglycemia (objectively measured fasting glucose ≥100 mg/dL, self-reported antidiabetic medication use, or self-reported physician-diagnosed diabetes).

CKD was defined by objectively measured elevated urinary albumin-to-creatinine ratio (UACR) (≥30 mg/g) or reduced estimated glomerular filtration rate (eGFR) (<60 mL/min/1.73 m^2^) (27), calculated using the 2009 Chronic Kidney Disease Epidemiology Collaboration (CKD-EPI) equation (28).

### 2.4 Covariates

Covariates encompassed three categories: (1) demographic characteristics (age [<45, 45–64, ≥65 years], sex, race/ethnicity [Non-Hispanic White, Non-Hispanic Black, Mexican American, Other Hispanic, Other Races]); (2) socioeconomic indicators (education [<high school, high school graduate, college or above], poverty-to-income ratio (PIR) [<1.3, 1.3–3.5, ≥3.5], marital status [married/cohabiting, divorced/separated/widowed, never married]); and (3) lifestyle factors. For lifestyle factors, smoking status was categorized as never smoker (smoked <100 cigarettes in lifetime), former smoker (smoked ≥100 cigarettes in lifetime but currently not smoking), or current smoker (smoked ≥100 cigarettes in lifetime and currently smoking). Participants’ alcohol intake was assessed through 24-h dietary recall interviews. We classified alcohol consumption into three categories: none (0 g/day), moderate intake (men: 0.1–27.9 g/day; women: 0.1–13.9 g/day), and heavy consumption (men: ≥28 g/day; women: ≥14 g/day). Body mass index (BMI) was calculated as weight (kg)/height (m)^2^. Participants were classified as normal weight (BMI < 25 kg/m^2^), overweight (BMI 25–29.9 kg/m^2^), or obese (BMI ≥ 30 kg/m^2^). Physical activity was assessed using the Global Physical Activity Questionnaire (GPAQ), which calculated total physical activity by summing the metabolic equivalent of task (MET)-minutes per week across three domains: (1) work-related activities (including both moderate and vigorous-intensity activities), (2) transportation (walking or bicycling), and (3) recreational activities (including both moderate and vigorous-intensity activities). According to WHO guidelines (29), physical activity levels were categorized as insufficient (<600 MET-minutes/week) or sufficient (≥600 MET-minutes/week).

### 2.5 Statistical analysis

Statistical analyses incorporated NHANES complex survey design elements. Sampling weights were calculated by multiplying 2-year SAF weights by 1/6 to account for the 12-year study period. Descriptive statistics presented weighted means with standard errors (SE) for continuous variables and weighted percentages for categorical variables. Between-group comparisons were conducted using weighted Student’s t-tests for continuous variables and chi-square tests for categorical variables.

Confounders were selected based on their association with CKM syndrome (*p* < 0.1) or if they changed the E-DII effect estimate by >10%. Sex was included based on established importance regardless of statistical criteria. The complete selection process is detailed in Supplementary Tables 1–4. We used multiple logistic regression to examine how E-DII scores relate to CKM syndrome. Three models were applied: Model 1 (no adjustments), Model 2 (adjusted for age, sex, and race/ethnicity), and Model 3 (further adjusted for education, poverty-to-income ratio, marital status, smoking, and physical activity). We analyzed E-DII both as a continuous variable and as quartiles, with the lowest quartile (Q1) serving as the reference. P-for-trend values were calculated to assess dose-response relationships. To further examine the dose-response relationship between E-DII scores and CKM syndrome, restricted cubic spline analyses were performed with four knots, adjusting for all covariates. To evaluate the robustness of the primary findings, stratified analyses were performed across population subgroups defined by age, sex, race/ethnicity, education level, marital status, poverty-to-income ratio, BMI, and alcohol consumption. Potential effect modification was evaluated by testing interactions between E-DII scores and each stratification variable. Results were presented as odds ratio (OR) with 95% CI.

To identify which specific dietary components contribute most to CKM syndrome, we used two complementary analytical approaches: Weighted Quantile Sum (WQS) regression and Quantile G-Computation (QGC). WQS regression helps us understand how different dietary components collectively affect CKM syndrome. It assigns positive weights to each component (all between 0 and 1, summing to 1) to show their relative importance. We then analyzed this weighted dietary index using logistic regression, adjusting for confounders. QGC allows components to have either positive or negative effects on CKM syndrome. Weights range from −1 to 1, helping us identify both harmful and beneficial dietary factors. We divided the resulting mixture index into quartiles and analyzed it using adjusted logistic regression.

To address potential bias from missing covariates, we performed multiple imputation using the chained equation approach with 5 replications in R. This method was applied to account for missing data on education level, marital status, PIR, smoking status, and physical activity. The imputation model included all covariates, exposure and outcome variables. We compared results from multiple imputation analysis (*n* = 10,293) with complete case analysis (*n* = 7,110) to evaluate finding robustness. Second, to assess potential selection bias, we compared characteristics between the final study population (*n* = 7,110) and participants excluded due to missing E-DII (*n* = 637) or CKM (*n* = 746) data. Standardized differences were calculated to quantify the magnitude of differences between groups, with values <0.1 indicating negligible differences. Chi-square tests for categorical variables and t-tests for continuous variables were performed to assess statistical significance. For participants with missing CKM data, the assessment of CMS and CKD components was based on available data, resulting in smaller sample sizes for these comparisons as indicated in the table. Unweighted data were used in this sensitivity analysis to directly assess differences between groups. Third, E-value analysis quantified the minimum strength needed for unmeasured confounders to nullify our observed E-DII and CKM syndrome associations (30). This method assessed both exposure-confounder and outcome-confounder relationships, with larger values indicating findings more resistant to unmeasured confounding.

Statistical analyses were conducted using R software (version 4.4.1, R Foundation for Statistical Computing, Vienna, Austria) and Empower Stats (X&Y Solutions Incorporated, Boston, MA, USA). Two-sided tests were employed with statistical significance set at *p* < 0.05.

## 3 Results

### 3.1 Baseline characteristics

Our analysis included 7,110 participants, of which 50.10% were male. The ranges of E-DII for quartiles 1–4 were −2.69 to −0.06, −0.06 to 0.58, 0.58–1.37, and 1.37–6.96, respectively, with a weighted mean (± SE) of 0.68 ± 0.02. Notably, 72.6% of participants (*n* = 5,160) had a pro-inflammatory diet (E-DII > 0), while 27.4% (*n* = 1,950) maintained an anti-inflammatory diet (E-DII < 0). In total, participants had a weighted prevalence of CKM syndrome of 3.50 ± 0.20%, and the prevalence increased with E-DII quartiles (Quartile 1: 2.67 ± 0.43%; Quartile 2: 2.98 ± 0.42%; Quartile 3: 2.70 ± 0.46%; Quartile 4: 5.97 ± 0.63%, *p* < 0.0001). The components of CKM syndrome also showed significant differences across quartiles, with CKD prevalence ranging from 4.80 to 8.71% (*p* < 0.0001) and CMS prevalence from 31.67 to 41.06% (*p* = 0.0008). We found statistically significant differences in demographic characteristics (sex, age, race/ethnicity), socioeconomic indicators (education, marital status, PIR), lifestyle factors (smoking status, alcohol consumption, BMI, physical activity), clinical measurements (waist circumference, diastolic blood pressure, UACR, eGFR), and most components of CMS including central obesity, low HDL-C, and hypertension (all *p* < 0.05) among E-DII quartiles (refer to Table 1 and Supplementary Table 5).

**Table 1:** Baseline characteristics of study population, weighted.

Characteristics	Overall (*N* = 7,110)
E-DII	0.68 ± 0.02
Demographic characteristics	
Sex, %(SE)	
Male	50.10 (0.65)
Female	49.90 (0.65)
Age group (years), %(SE)	
<45	46.39 (1.03)
45–64	37.39 (0.94)
≥65	16.21 (0.63)
Race/ethnicity, %(SE)	
Mexican American	7.25 (0.66)
Other Hispanic	4.88 (0.46)
Non-Hispanic White	70.95 (1.43)
Non-Hispanic Black	9.75 (0.74)
Other Race	7.17 (0.46)
Socioeconomic indicators	
Education level, %(SE)	
<High school	12.29 (0.70)
High school	21.10 (0.92)
>High school	66.61 (1.22)
Marital status, %(SE)	
Married/Living with a partner	66.02 (0.97)
Divorced/Separated/Widowed	15.97 (0.66)
Never married	18.00 (0.81)
Poverty-to-income ratio, %(SE)	
<1.3	18.67 (0.86)
≥1.3, <3.5	35.38 (0.97)
≥3.5	45.95 (1.26)
Lifestyle factors	
Smoking status, %(SE)	
Never	56.61 (1.06)
Former	26.41 (0.92)
Current	16.98 (0.67)
Alcohol consumption, %(SE)	
None	75.16 (0.82)
Moderate intake	8.87 (0.46)
Heavy consumption	15.97 (0.68)
BMI (kg/m^2^), %(SE)	
<25	31.17 (0.88)
≥25, <30	33.80 (0.70)
≥30	35.04 (0.84)
Physical activity (MET-min/week), %(SE)	
<600	17.29 (0.56)
≥600	82.71 (0.56)
Clinical measurements	
WC (cm)	98.55 ± 0.31
TG (mg/dL)	118.91 ± 1.46
HDL-C (mg/dL)	54.67 ± 0.30
FPG (mg/dL)	104.75 ± 0.44
Mean SBP (mmHg)	120.42 ± 0.30
Mean DBP (mmHg)	70.02 ± 0.27
UACR (mg/g)	2.37 ± 0.21
eGFR (mL/min/1.73 m^2^)	95.72 ± 0.43
Components and outcomes	
Central obesity, %(SE)	54.97 (1.00)
Hypertriglyceridemia, %(SE)	22.67 (0.73)
Low HDL-C, %(SE)	25.36 (0.78)
Hypertension, %(SE)	43.11 (1.00)
Hyperglycemia, %(SE)	50.26 (0.99)
CMS, %(SE)	35.86 (0.84)
CKD, %(SE)	5.58 (0.28)
CKM, %(SE)	3.50 (0.20)

Data are presented as weighted means ± standard error (SE) for continuous variables and weighted percentages (SE) for categorical variables. Complete data for all quartiles are available in Supplementary Table 1.

E-DII, energy-adjusted dietary inflammatory index; BMI, body mass index; MET, metabolic equivalent of task; WC, waist circumference; TG, triglycerides; HDL-C, high-density lipoprotein cholesterol; FPG, fasting plasma glucose; SBP, systolic blood pressure; DBP, diastolic blood pressure; UACR, urinary albumin-to-creatinine ratio; eGFR, estimated glomerular filtration rate; CMS, cardiometabolic syndrome; CKD, chronic kidney disease; CKM, cardiovascular-kidney-metabolic syndrome.

### 3.2 The relationship between E-DII and CKM syndrome and its components

Table 2 presents the associations between E-DII and CKM syndrome and its components. In the fully adjusted model (Model 3), each unit increase in E-DII score was significantly associated with higher odds of CKM syndrome (OR = 1.22, 95% CI: 1.09–1.37). Participants in the highest E-DII quartile (most pro-inflammatory diet) had 2.07 times higher odds of CKM syndrome compared to those in the lowest quartile (most anti-inflammatory diet) (OR = 1.22, 95% CI: 1.29–3.31; *p* for trend = 0.0040).

**Table 2:** Associations between energy-adjusted dietary inflammatory index (E-DII) and CKM syndrome and its components: results from multivariate logistic regression models, weighted.

Energy-adjusted dietary inflammatory index	Crude model (Model 1)	Minimally adjusted model (Model 2)	Fully adjusted model (Model 3)
CKM syndrome/OR (95% CI)			
Continuous	1.28 (1.18, 1.40)	1.27 (1.14, 1.40)	1.22 (1.09, 1.37)
Categories			
Quartile 1	Reference	Reference	Reference
Quartile 2	1.12 (0.71, 1.77)	1.31 (0.81, 2.14)	1.24 (0.76, 2.04)
Quartile 3	1.01 (0.64, 1.61)	1.24 (0.77, 2.01)	1.13 (0.69, 1.85)
Quartile 4	2.31 (1.61, 3.33)	2.38 (1.54, 3.68)	2.07 (1.29, 3.31)
*p* for trend	<0.0001	0.0002	0.0040
CKD/OR (95% CI)			
Continuous	1.23 (1.14, 1.33)	1.22 (1.12, 1.32)	1.17 (1.07, 1.29)
Categories			
Quartile 1	Reference	Reference	Reference
Quartile 2	0.90 (0.62, 1.30)	1.04 (0.69, 1.55)	0.99 (0.66, 1.48)
Quartile 3	1.03 (0.71, 1.49)	1.29 (0.86, 1.92)	1.18 (0.79, 1.79)
Quartile 4	1.89 (1.44, 2.49)	1.96 (1.41, 2.71)	1.71 (1.21, 2.42)
*p* for trend	<0.0001	<0.0001	0.0020
CMS syndrome/OR (95% CI)			
Continuous	1.13 (1.07, 1.19)	1.19 (1.13, 1.26)	1.14 (1.07, 1.21)
Categories			
Quartile 1	Reference	Reference	Reference
Quartile 2	1.25 (1.07, 1.45)	1.38 (1.19, 1.61)	1.31 (1.13, 1.51)
Quartile 3	1.15 (0.97, 1.37)	1.33 (1.11, 1.59)	1.21 (1.01, 1.45)
Quartile 4	1.50 (1.24, 1.82)	1.82 (1.50, 2.20)	1.57 (1.28, 1.92)
*p* for trend	0.0008	<0.0001	0.0005
Central obesity/OR (95% CI)			
Continuous	1.20 (1.14, 1.27)	1.14 (1.07, 1.21)	1.13 (1.06, 1.20)
Categories			
Quartile 1	Reference	Reference	Reference
Quartile 2	1.34 (1.12, 1.60)	1.39 (1.15, 1.67)	1.37 (1.13, 1.66)
Quartile 3	1.42 (1.18, 1.73)	1.39 (1.13, 1.70)	1.38 (1.12, 1.69)
Quartile 4	1.79 (1.49, 2.16)	1.52 (1.25, 1.84)	1.48 (1.21, 1.81)
*p* for trend	<0.0001	0.0001	0.0005
Hypertriglyceridemia/OR (95% CI)			
Continuous	1.07 (1.01, 1.14)	1.18 (1.10, 1.25)	1.13 (1.06, 1.20)
Categories			
Quartile 1	Reference	Reference	Reference
Quartile 2	1.09 (0.88, 1.35)	1.17 (0.94, 1.46)	1.10 (0.88, 1.37)
Quartile 3	1.18 (0.95, 1.46)	1.38 (1.10, 1.74)	1.25 (0.99, 1.57)
Quartile 4	1.20 (0.98, 1.48)	1.61 (1.29, 2.00)	1.38 (1.10, 1.73)
*p* for trend	0.0595	<0.0001	0.0047
Low HDL-C/OR (95% CI)			
Continuous	1.20 (1.14, 1.26)	1.19 (1.12, 1.26)	1.13 (1.07, 1.20)
Categories			
Quartile 1	Reference	Reference	Reference
Quartile 2	1.41 (1.20, 1.65)	1.39 (1.18, 1.64)	1.30 (1.10, 1.53)
Quartile 3	1.30 (1.12, 1.52)	1.28 (1.10, 1.50)	1.14 (0.97, 1.34)
Quartile 4	1.90 (1.57, 2.29)	1.84 (1.52, 2.23)	1.55 (1.27, 1.89)
*p* for trend	<0.0001	<0.0001	0.0005
Hypertension/OR (95% CI)			
Continuous	1.06 (1.01, 1.11)	1.14 (1.08, 1.21)	1.09 (1.03, 1.16)
Categories			
Quartile 1	Reference	Reference	Reference
Quartile 2	1.04 (0.90, 1.22)	1.21 (1.01, 1.44)	1.14 (0.95, 1.36)
Quartile 3	1.03 (0.88, 1.20)	1.25 (1.05, 1.48)	1.13 (0.95, 1.35)
Quartile 4	1.21 (1.04, 1.40)	1.58 (1.34, 1.88)	1.36 (1.14, 1.64)
*p* for trend	0.0408	<0.0001	0.0056
Hyperglycemia/OR (95% CI)			
Continuous	0.98 (0.93, 1.03)	1.10 (1.04, 1.16)	1.07 (1.01, 1.13)
Categories			
Quartile 1	Reference	Reference	Reference
Quartile 2	1.01 (0.85, 1.19)	1.16 (0.97, 1.37)	1.11 (0.94, 1.32)
Quartile 3	0.88 (0.73, 1.05)	1.12 (0.92, 1.35)	1.06 (0.87, 1.28)
Quartile 4	0.94 (0.78, 1.12)	1.37 (1.13, 1.66)	1.26 (1.03, 1.53)
*p* for trend	0.2559	0.0060	0.0571

Model 1: Unadjusted model without covariates.

Model 2: Minimally adjusted model controlling for sex, age and race.

Model 3: Fully adjusted model controlling for sex, age, race, education level, marital status, poverty-to-income ratio, smoking status and physical activity measured in total METs per week.

E-DII, energy-adjusted dietary inflammatory index; CKM, cardiovascular-kidney-metabolic syndrome; CKD, chronic kidney disease; CMS, cardiometabolic syndrome; HDL-C, high-density lipoprotein cholesterol; OR, odds ratio; CI, confidence interval; MET, metabolic equivalent of task.

For the primary components of CKM syndrome, each unit increase in E-DII was significantly associated with higher odds of both CKD (OR = 1.17, 95% CI: 1.07–1.29) and CMS (OR = 1.14, 95% CI: 1.07–1.21) in the fully adjusted models. Categorical analysis revealed that participants in the highest E-DII quartile had 1.71 times higher odds of CKD (OR = 1.17, 95% CI: 1.21–2.42; *p* for trend = 0.0020) and 1.57 times higher odds of CMS (OR = 1.57, 95% CI: 1.28–1.92; *p* for trend = 0.0005) compared to the lowest quartile.

Among the individual components of CMS, a pro-inflammatory diet (higher E-DII) was significantly associated with increased odds of all five components in the fully adjusted models. The strongest associations were observed for central obesity (OR = 1.13, 95% CI: 1.06–1.20), hypertriglyceridemia (OR = 1.13, 95% CI: 1.06–1.20), and low HDL-C (OR = 1.13, 95% CI: 1.07–1.20), followed by hypertension (OR = 1.09, 95% CI: 1.03–1.16) and hyperglycemia (OR = 1.07, 95% CI: 1.01–1.13).

### 3.3 Analysis of restricted cubic spline regression

**Figure 2:**
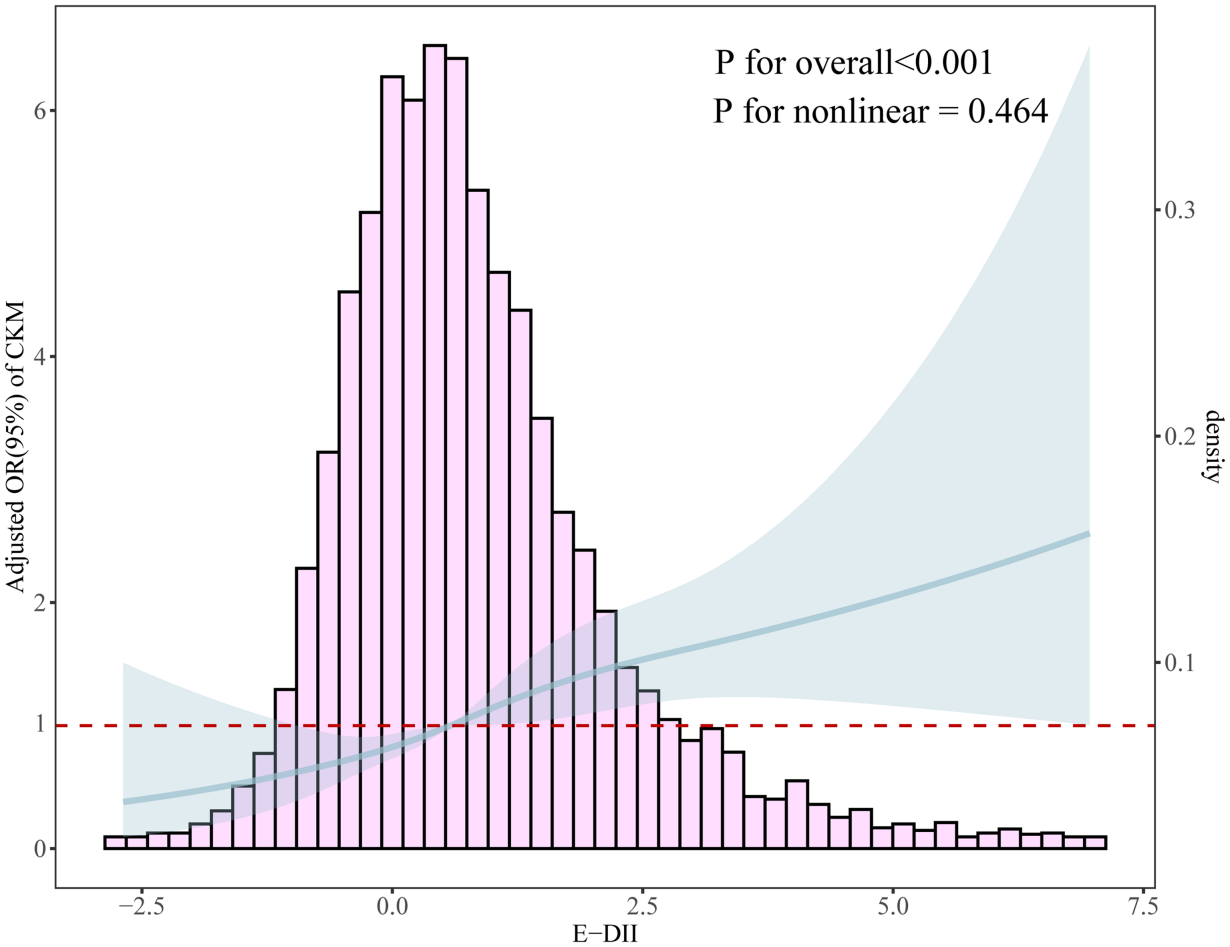
Dose-response relationship between E-DII and CKM syndrome using restricted cubic spline analysis with four knots (*p* for overall < 0.001, *p* for nonlinearity = 0.464). The solid line represents the adjusted odds ratios from the fully adjusted model (Model 3), and the shaded area represents the 95% confidence intervals. The histogram shows the distribution of E-DII scores in the study population. The non-significant *p* for nonlinearity indicates a linear relationship between E-DII and CKM syndrome.

As shown in Figure 2, a significant positive association was observed between E-DII scores and CKM syndrome (*p* for overall < 0.001). The relationship was linear (*p* for nonlinearity = 0.464).

### 3.4 Subgroup analysis and interaction

**Figure 3:**
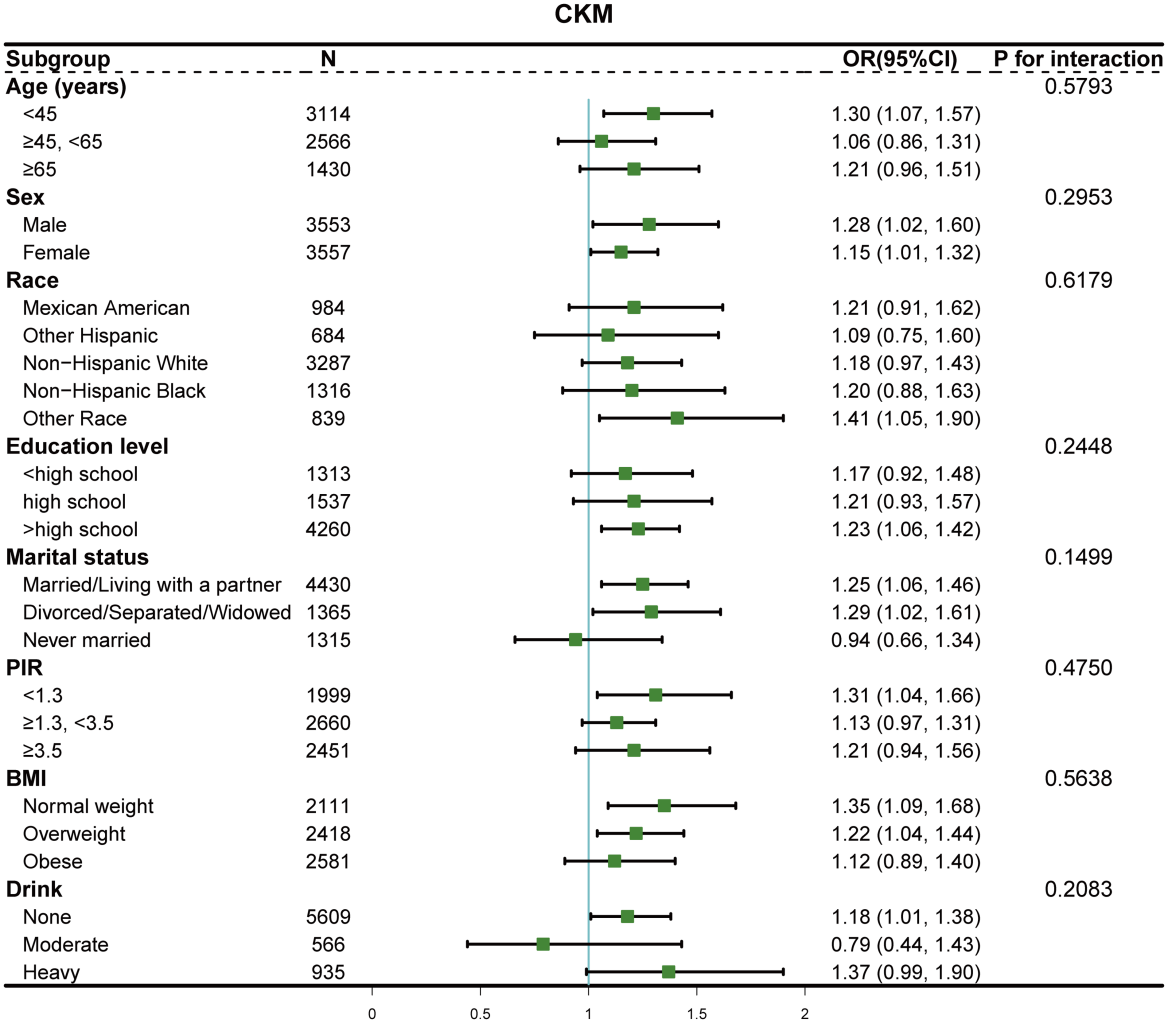
Forest plot of stratified analyses examining E-DII and CKM syndrome associations by demographic and socioeconomic characteristics. The odds ratios (dots) and 95% confidence intervals (horizontal lines) were adjusted for confounding factors, excluding the stratification variable in each subgroup. *p* for interaction was calculated for each stratification.

To evaluate the consistency of associations between E-DII and CKM syndrome across population characteristics, we performed stratified analyses. As shown in Figure 3, no significant effect modifications were observed for any stratification variables (all *p* for interaction > 0.05). The positive associations were consistently observed and remained statistically significant in several subgroups, including adults aged <45 years, both males and females, other races, those with higher education level, married/living with partner or divorced/separated/widowed individuals, and those with lower poverty-to-income ratio (<1.3). When stratifying by BMI categories, the association was significant among normal weight [OR = 1.35 (95% CI, 1.09–1.68)] and overweight individuals [OR = 1.22 (95% CI, 1.04–1.44)], but not in the obese group [OR = 1.12 (95% CI, 0.89–1.40)]. For alcohol consumption, significant association was observed among non-drinkers [OR = 1.18 (95% CI, 1.01–1.38)], with a stronger but marginally significant association among heavy drinkers [OR = 1.37 (95% CI, 0.99–1.90)].

### 3.5 Alcohol consumption as the primary inflammatory dietary factor associated with CKM syndrome

**Figure 4:**
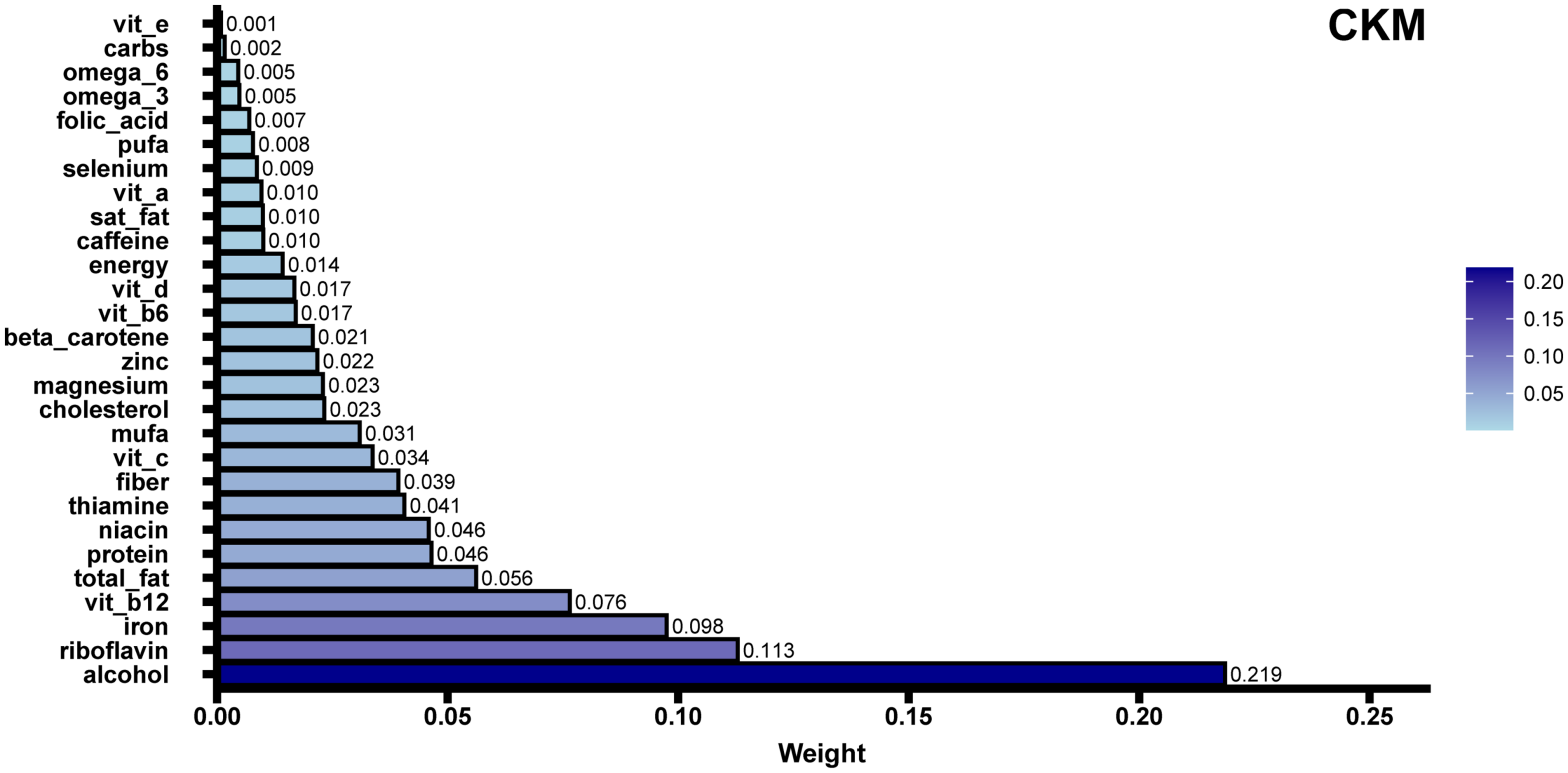
WQS regression analysis of E-DII components in relation to CKM syndrome. The bar plot shows the relative contribution (weights) of 28 dietary components to CKM syndrome risk, with the sum of all weights constrained to 1. Longer bars indicate stronger associations with CKM syndrome, with alcohol showing the highest weight (0.219). WQS regression helps identify which specific dietary components contribute most to CKM syndrome risk while accounting for the complex correlations among components. For each unit increase in the weighted index, participants had 4.62 times higher odds of CKM syndrome (OR = 4.62, 95% CI: 1.95–11.02, *p* = 0.0005). The model was adjusted for potential confounders including sex, age, race, education level, marital status, poverty-to-income ratio, smoking status, and physical activity.

**Figure 5:**
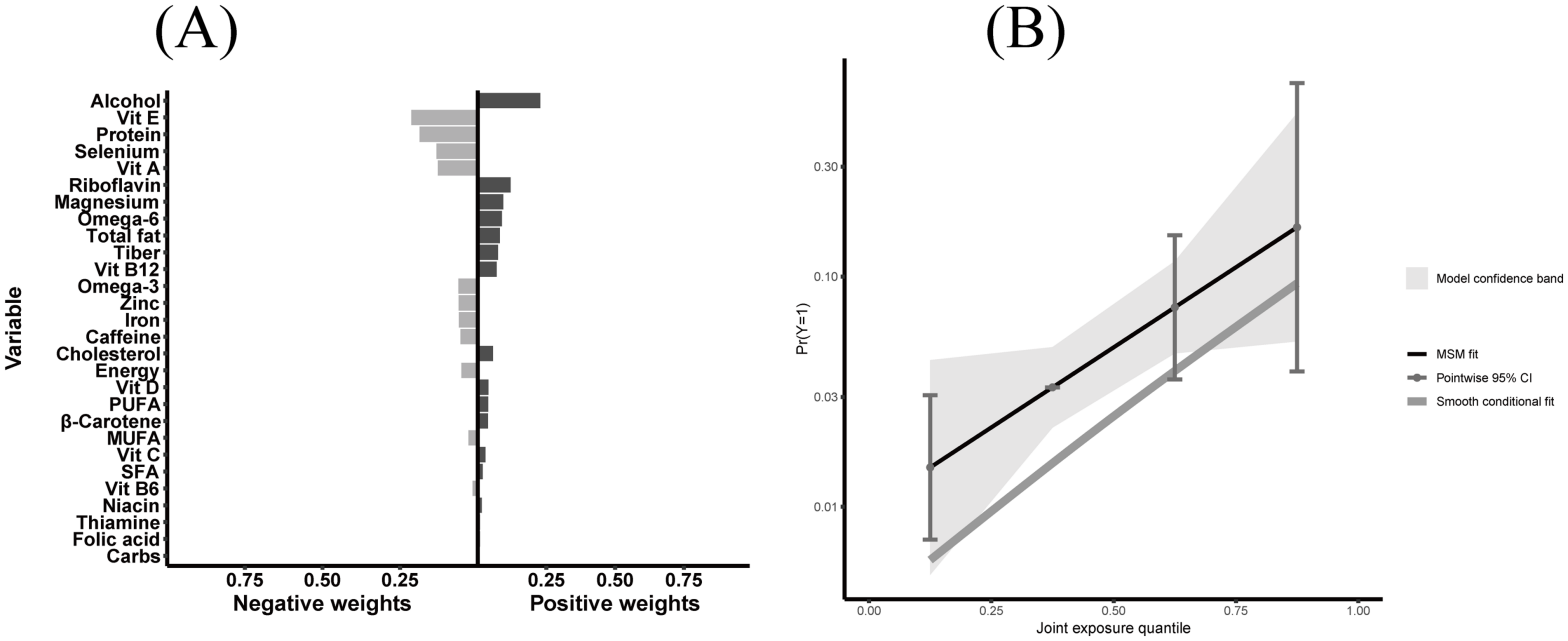
Quantile G-computation (QGC) analysis of E-DII components in relation to CKM syndrome. **(A)** Component-specific weights showing positive (right, pro-inflammatory effect) and negative (left, anti-inflammatory effect) contributions of 28 E-DII components to CKM syndrome, with alcohol having the highest positive weight. **(B)** The relationship between joint exposure quantiles of E-DII components and predicted probability of CKM syndrome, with marginal structural model fit (solid line), 95% confidence intervals (shaded area), and smooth conditional fit (dashed line). Participants in the highest quartile of the mixture index had 2.23 times the odds of CKM syndrome compared to those in the lowest quartile (OR = 2.23, 95% CI: 1.08–4.58, *p* = 0.030). Both panels were adjusted for potential confounders including sex, age, race, education level, marital status, poverty-to-income ratio, smoking status, and physical activity.

To identify dietary components associated with CKM syndrome, we performed both WQS regression and QGC analysis. Among the 28 DII components, WQS regression identified alcohol as having the highest weight (weight = 0.219) (Figure 4). This finding was supported by the QGC analysis, where alcohol also demonstrated the highest positive weight (Figure 5A). The consistency between these two methods highlighted alcohol as the dietary component most strongly associated with CKM syndrome. Both methods confirmed the positive association between inflammatory dietary components and CKM syndrome. In terms of the overall association strength, the WQS regression showed that for each unit difference in the weighted index, the odds of CKM syndrome were 4.62 times that of the reference level (OR = 4.62, 95% CI: 1.95–11.02, *p* = 0.0005). The QGC analysis showed that participants in the higher quartile of the mixture index had 2.23 times the odds of CKM syndrome compared to those in the lower quartile (OR = 2.23, 95% CI: 1.08–4.58, *p* = 0.030), as illustrated in Figure 5B.

### 3.6 Sensitivity analysis

Sensitivity analyses using multiple imputation for missing covariates (*n* = 10,293) yielded results consistent with the complete case analysis (*n* = 7,110). In the multiple imputation analysis, each unit increase in E-DII was associated with a 24% higher prevalence of CKM syndrome (OR = 1.24, 95% CI: 1.14–1.35, *p* < 0.001), while participants in the highest E-DII quartile exhibited a 116% higher prevalence compared to those in the lowest quartile (OR = 2.16, 95% CI: 1.52–3.08, *p* < 0.001). Similar significant associations were consistently observed across all CKM syndrome components (refer to Supplementary Table 6). Second, participants excluded due to missing E-DII or CKM data differed from the final study population in demographic, socioeconomic, lifestyle, and clinical characteristics. However, the prevalence of CKM syndrome, the primary outcome, was comparable across groups, suggesting minimal impact of selection bias on the study conclusions (detailed in Supplementary Table 7). E-value analysis showed the associations between E-DII and CKM syndrome, as well as its components, were robust to unmeasured confounding. For CKM syndrome, the E-value was 2.23 (CI limit: 1.53), with secondary outcomes and components showing similarly high values (≥1.49), supporting the strength of the findings (refer to Supplementary Table 8).

## 4 Discussion

Our analyses demonstrated significant associations between dietary inflammatory potential and CKM syndrome (OR = 1.22, 95% CI: 1.09–1.37). The relationship exhibited a linear dose-response pattern (*p* for nonlinearity = 0.464), suggesting a consistent effect of dietary inflammatory potential on CKM syndrome risk. Similar associations were observed for both major components: chronic kidney disease (OR: 1.17, 95% CI: 1.07–1.29) and cardiometabolic syndrome (OR: 1.14, 95% CI: 1.07–1.21).

Our stratified analysis revealed a gradient pattern in the E-DII and CKM syndrome association across BMI categories, with the strongest relationship observed among normal weight individuals, followed by overweight participants, while becoming attenuated and non-significant in the obese group. This pattern aligns with emerging evidence suggesting that normal weight individuals may operate within a distinct metabolic environment where dietary inflammation plays a more prominent role in disease pathogenesis, without the confounding effects of obesity-induced chronic inflammation (31, 32). In obese individuals, the baseline inflammatory state accompanying excess adiposity may obscure the additional inflammatory burden from dietary factors (33). These findings highlight the potential importance of targeting dietary inflammation particularly among normal weight individuals who might otherwise be considered at lower risk based solely on BMI criteria.

Our stratified analysis by alcohol consumption revealed varying patterns in the association between E-DII and CKM syndrome across drinking categories, with significant associations among non-drinkers, a stronger trend among heavy drinkers, and no significant association among moderate drinkers. This differential pattern addresses an important question regarding whether moderate alcohol intake has different effects compared to high consumption in relation to CKM syndrome. Indeed, our results suggest that moderate alcohol consumption may potentially modify the inflammatory impact of diet on CKM syndrome risk differently than either abstention or heavy drinking. This finding is particularly intriguing considering our component analysis identified alcohol as one of the most influential dietary factors contributing to the inflammatory potential of diet in relation to CKM syndrome.

The differential associations may reflect complex interactions between alcohol and other dietary components in modulating systemic inflammation. For non-drinkers, the inflammatory potential of the remaining dietary components significantly associated with CKM syndrome risk. The trend toward a stronger association among heavy drinkers is consistent with research demonstrating that excessive alcohol consumption can enhance pro-inflammatory processes through mechanisms such as alteration of monocyte function with increased cytokine production (34), disruption of gut barrier integrity (35, 36), and activation of systemic inflammatory responses (37). In contrast, the absence of significant association among moderate drinkers might potentially reflect the counterbalancing effects of moderate alcohol consumption on inflammatory pathways, as studies have shown moderate intake can down-regulate inflammatory mediators like NF-κB (38) and reduce levels of inflammatory markers such as C-reactive protein and interleukin-6 (39). However, these interpretations warrant caution due to the smaller sample sizes in some categories and the non-significant interaction test.

To identify which dietary factors contribute most to CKM syndrome, we used two advanced statistical methods (WQS regression and QGC analysis). These methods account for the complex correlations among dietary components while controlling for potential confounders. Our analysis identified alcohol consumption as a major contributor to CKM syndrome. This highlights the potential importance of reducing alcohol intake in populations at risk of CKM syndrome.

This study adds to the current knowledge regarding dietary inflammatory potential in relation to cardiorenal and metabolic disorders. Multiple epidemiological studies confirm that individuals with higher DII scores exhibit elevated risks of both cardiovascular incidence and mortality. Yuan et al. (40) demonstrated that participants in the highest DII quartile had a 37% increased risk of cardiovascular mortality compared to those in the lowest quartile, with this association remaining robust across different baseline glycemic statuses. This dose-response relationship was further corroborated by Shivappa et al.’s meta-analysis (41), which revealed that each one-point increase in DII score was associated with an 8% increase in cardiovascular mortality risk. Regarding kidney function, Xiao et al. (42) found that higher DII was significantly associated with CKD progression, while Bondonno et al. (43) reported associations between elevated DII scores and poorer kidney function in elderly populations. Recent work by Ding et al. (44) identified a significant relationship between DII and albuminuria, suggesting inflammation’s role in nephron damage and kidney function deterioration. These findings align with previous meta-analyses demonstrating that subjects in the highest DII exposure category exhibited a 44% greater risk of developing CKD compared with those in the lowest category (RR: 1.44; 95% CI: 1.22–1.71) (18). For metabolic outcomes, Bakhshimoghaddam et al. (45) confirmed a significant correlation between higher DII scores and increased MetS risk across multiple observational studies. Namazi et al. (46) demonstrated that elevated DII scores correlate with increased blood pressure and triglycerides, while Guo et al. (47) noted associations with low HDL cholesterol and hyperglycemia in middle-aged and older populations. These findings support previous meta-analyses showing that subjects with pro-inflammatory dietary patterns demonstrated significantly increased risks of MetS (OR: 1.23; 95% CI: 1.10–1.37) and its components (20). While these studies focused on individual components, our investigation extends this research by examining CKM syndrome as an integrated entity, providing evidence that dietary inflammatory potential may influence the syndrome as a whole.

The identified association between alcohol consumption and CKM syndrome is supported by multiple organ-specific molecular mechanisms. In the cardiovascular system, alcohol exhibits a characteristic U-shaped relationship with disease risk. Moderate consumption may confer protective effects through modulation of biological markers, including increased HDL cholesterol and decreased fibrinogen levels (48, 49). However, excessive intake promotes cardiovascular damage through direct myocardial toxicity, mitochondrial dysfunction, and oxidative stress (50, 51). Regarding renal function, alcohol affects the kidney through both direct nephrotoxicity and indirect hemodynamic alterations. Chronic alcohol consumption impairs renal tubular function and contributes to kidney damage through generation of reactive oxygen species, reduced antioxidant capacity, and activation of pro-inflammatory cytokines (52, 53). The relationship between alcohol intake and CKD risk shows a similar pattern to that observed with cardiovascular outcomes (54), where excessive consumption clearly increases disease risk through specific pathways related to oxidative stress and inflammatory cytokine activation (55). Metabolic dysregulation from alcohol consumption occurs through distinct mechanisms including disruption of hepatic glucose metabolism, pancreatic function, and adipose tissue regulation (56, 57). Non-alcoholic fatty liver disease, often considered a liver manifestation of metabolic syndrome, shares common pathways with alcohol-related liver damage, where insulin resistance and inflammation play pivotal roles (57, 58). Alcohol exacerbates fat accumulation in the liver, especially when combined with metabolic risk factors, leading to increased likelihood of developing conditions that influence both cardiovascular and renal health (59).

This study has several methodological strengths. The application of both WQS regression and QGC analysis provides robust evidence for the role of specific dietary components in CKM syndrome. The consistency of associations across demographic and socioeconomic subgroups supports the reliability of these findings. However, limitations should be considered. The 24-h dietary recall method may not fully capture long-term dietary patterns and is subject to recall bias and seasonal variations in food consumption. These limitations of the dietary assessment method may lead to misclassification of participants’ true inflammatory dietary exposure, potentially changing the observed associations between E-DII and CKM syndrome components. Additionally, the E-DII assumes linear relationships between dietary components and inflammatory markers, which may not capture complex dose-response patterns such as U-shaped associations demonstrated in our alcohol stratification analysis. The cross-sectional design prevents establishment of temporal relationships between dietary factors and CKM syndrome, therefore causality cannot be inferred from these associations. Despite using multiple imputation for covariates, missing data in population selection, E-DII calculation, and CKM syndrome assessment could not be imputed. This reduced our analytic sample, potentially limiting its representativeness of the original NHANES dataset, restricting generalizability to the U.S. population, and affecting survey weight validity. Despite adjustment for confounders, several unmeasured factors may still influence our findings, including genetic susceptibility to dietary inflammatory responses, unreported dietary supplement use, and long-term dietary pattern changes not captured by the 24-h recall method. Furthermore, NHANES data excludes institutionalized individuals and those with extreme dietary habits, and the findings may not be generalizable to populations outside the U.S. due to differences in dietary patterns and CKM syndrome risk factors.

These results have important implications for clinical practice, public health strategies, and dietary guidelines. The identification of alcohol as a key dietary component suggests that targeted interventions focusing on alcohol consumption are highly relevant for CKM syndrome prevention and management. For clinical practice, healthcare providers should incorporate alcohol consumption assessment into routine care for patients at risk of CKM syndrome and develop individualized alcohol reduction plans for diagnosed patients. From a public health perspective, strategies should include awareness campaigns highlighting the link between alcohol and CKM syndrome, community education on healthy drinking limits, and policy measures to reduce excessive alcohol consumption. Current dietary guidelines may need revision to emphasize stricter alcohol limits for individuals at high risk for CKM syndrome, particularly those with existing cardiorenal or metabolic abnormalities. Future research directions should include longitudinal studies to establish temporal relationships, intervention studies to evaluate the impact of alcohol reduction on CKM syndrome progression, and investigation of potential interaction effects between alcohol and other dietary components in CKM syndrome development.

## 5 Conclusion

The present analysis revealed significant associations between dietary inflammatory potential and CKM syndrome among US adults, with alcohol consumption emerging as the primary contributing dietary factor. The observed dose-response relationship between E-DII scores and CKM syndrome suggests that dietary patterns, particularly alcohol intake, may represent modifiable targets for CKM syndrome prevention strategies. While these cross-sectional data demonstrate associations between dietary inflammatory potential and CKM syndrome, prospective studies are needed to elucidate temporal relationships.

## Data Availability

Publicly available datasets were analyzed in this study. This data can be found here: the NHANES data used in this study are publicly available from the Centers for Disease Control and Prevention (CDC) website: https://wwwn.cdc.gov/nchs/nhanes/Default.aspx Repository: National Center for Health Statistics (NCHS) no accession numbers are required for accessing these public datasets.

## Glossary

CDC: Centers for Disease Control and Prevention
CI: confidence interval
CKD: chronic kidney disease
CKD-EPI: Chronic Kidney Disease Epidemiology Collaboration
CKM: cardiovascular-kidney-metabolic
CMS: cardiometabolic syndrome
CRP: C-reactive protein
CVD: cardiovascular disease
DBP: diastolic blood pressure
DII: Dietary Inflammatory Index
E-DII: energy-adjusted dietary inflammatory index
eGFR: estimated glomerular filtration rate
FPG: fasting plasma glucose
HDL-C: high-density lipoprotein cholesterol
HR: hazard ratio
IL-6: Interleukin-6
MET: metabolic equivalent of task
MetS: metabolic syndrome
MUFA: monounsaturated fatty acids
NCEP-ATP III: National Cholesterol Education Program Adult Treatment Panel III
NHANES: National Health and Nutrition Examination Survey
OR: odds ratio
PIR: poverty-to-income ratio
PUFA: polyunsaturated fatty acids
QGC: Quantile G-Computation
RR: relative risk
SBP: systolic blood pressure
SE: standard errors
SFA: saturated fatty acids
TG: triglycerides
UACR: urinary albumin-to-creatinine ratio
WC: waist circumference
WQS: Weighted Quantile Sum
