# Supplementary material for: Association between dietary inflammatory index score and cardiovascular-kidney-metabolic syndrome: a cross-sectional study based on NHANES
Source: Front Nutr. 2025 May 9;12:1557491. doi: 10.3389/fnut.2025.1557491 (PMC12098081; doi:10.3389/fnut.2025.1557491)
Supplement: Supplementary file 1 [file Table_1.DOCX]

**Supplementary Table 1: Collinearity Assessment of Potential Covariates Using Variance Inflation Factor (VIF)**

| **Variable** | **VIF** |
| --- | --- |
| E-DII | 1.1 |
| Sex | 1.1 |
| Age | 1.1 |
| Race/ethnicity | 1.1 |
| Education level | 1.3 |
| Marital status | 1.1 |
| Poverty-to-income ratio | 1.3 |
| Smoking status | 1.1 |
| Physical activity | 1.0 |

Note: VIF values < 5 indicate absence of substantial multicollinearity among the covariates.
