# Supplementary material for: Association between dietary inflammatory index score and cardiovascular-kidney-metabolic syndrome: a cross-sectional study based on NHANES
Source: Front Nutr. 2025 May 9;12:1557491. doi: 10.3389/fnut.2025.1557491 (PMC12098081; doi:10.3389/fnut.2025.1557491)
Supplement: Supplementary file 2 [file Table_2.DOCX]

**Supplementary Table 2: Univariate Associations Between Potential Covariates and CKM Syndrome**

| **Covariate** | **Category** | **β** | **SE** | **OR** | **95% CI** | **P-value** |
| --- | --- | --- | --- | --- | --- | --- |
| Sex | Female | 0.1394 | 0.1159 | 1.1496 | 0.9160-1.4427 | 0.2290 |
| Age | 45-64 | 1.5688 | 0.2463 | 4.8009 | 2.9624-7.7803 | <0.0001 |
|  | ≥65 | 3.2384 | 0.2313 | 25.4941 | 16.2022-40.1148 | <0.0001 |
| Race/ethnicity | Other Hispanic | -0.0609 | 0.2994 | 0.9409 | 0.5232-1.6921 | 0.8388 |
|  | Non-Hispanic White | 0.6635 | 0.2033 | 1.9415 | 1.3035-2.8917 | 0.0011 |
|  | Non-Hispanic Black | 0.4355 | 0.2308 | 1.5457 | 0.9832-2.4299 | 0.0592 |
|  | Other Race | -0.0745 | 0.2833 | 0.9282 | 0.5328-1.6172 | 0.7925 |
| Education level | High school | -0.0290 | 0.1661 | 0.9714 | 0.7015-1.3451 | 0.8612 |
|  | Above high school | -0.4031 | 0.1457 | 0.6683 | 0.5023-0.8891 | 0.0057 |
| Marital status | Divorced/Separated/Widowed | 0.5778 | 0.1261 | 1.7821 | 1.3920-2.2816 | <0.0001 |
|  | Never married | -1.3132 | 0.2620 | 0.2689 | 0.1609-0.4495 | <0.0001 |
| Poverty-to-income ratio | ≥1.3, <3.5 | 0.3096 | 0.1429 | 1.3628 | 1.0300-1.8032 | 0.0303 |
|  | ≥3.5 | -0.0895 | 0.1567 | 0.9144 | 0.6726-1.2431 | 0.5679 |
| Smoking status | Former | 0.6970 | 0.1233 | 2.0077 | 1.5766-2.5568 | <0.0001 |
|  | Current | -0.3779 | 0.1950 | 0.6853 | 0.4676-1.0043 | 0.0526 |
| Physical activity | Active | -0.8440 | 0.1242 | 0.4300 | 0.3371-0.5485 | <0.0001 |

Note: All analyses were based on a sample size of 7,110.

Reference categories - Sex: Male; Age:<45; Race/ethnicity: Mexican American; Education level: Below high school; Marital status: Married/Living with a partner; Poverty-to-income ratio: <1.3; Smoking status: Never smoker; Physical activity: Inactive.

**Abbreviations:** CKM, cardiovascular-kidney-metabolic syndrome; SE, standard error; OR: Odds Ratio; CI: Confidence Interval.
