# Supplementary material for: Association between dietary inflammatory index score and cardiovascular-kidney-metabolic syndrome: a cross-sectional study based on NHANES
Source: Front Nutr. 2025 May 9;12:1557491. doi: 10.3389/fnut.2025.1557491 (PMC12098081; doi:10.3389/fnut.2025.1557491)
Supplement: Supplementary file 3 [file Table_3.DOCX]

**Supplementary Table 3: Change in E-DII Coefficient with Addition or Removal of Covariates**

| **Covariate** | **Basic Model + Covariate** | **Full Model - Covariate** | **Selected** |
| --- | --- | --- | --- |
| Initial regression coefficient | 0.2505 | 0.2160 |  |
| Sex | 0.2562 | 0.2230 |  |
| Age | 0.2306 | 0.2434 * | Yes |
| Race/ethnicity | 0.2593 | 0.2093 |  |
| Education level | 0.2327 | 0.2200 |  |
| Marital status | 0.2415 | 0.2153 |  |
| Poverty-to-income ratio | 0.2499 | 0.2239 |  |
| Smoking status | 0.2770 * | 0.2136 | Yes |
| Physical activity | 0.2336 | 0.2140 |  |

Note: *: indicates >10% change in E-DII regression coefficient compared to the initial value. The basic model included only the exposure variable (E-DII), while the full model included all potential covariates.
