# Supplementary material for: Association between dietary inflammatory index score and cardiovascular-kidney-metabolic syndrome: a cross-sectional study based on NHANES
Source: Front Nutr. 2025 May 9;12:1557491. doi: 10.3389/fnut.2025.1557491 (PMC12098081; doi:10.3389/fnut.2025.1557491)
Supplement: Supplementary file 4 [file Table_4.DOCX]

**Supplementary Table 4: Final Covariate Selection for CKM Syndrome Model**

| **Outcome** | **Exposure** | **Selected by Criterion 1** | **Selected by Criterion 2** |
| --- | --- | --- | --- |
| CKM | E-DII | Age, Smoking status | Age, Race/ethnicity, Education level, Marital status, Poverty-to-income ratio, Smoking status, Physical activity |

Notes:

Criterion 1: Covariates that changed the exposure regression coefficient by >10% when added to the basic model or removed from the full model

Criterion 2: Covariates that met Criterion 1 or showed an association with the outcome at P<0.1

Final model included sex and all covariates selected by Criterion 2 to ensure comprehensive confounder adjustment
