# Supplementary material for: Association between dietary inflammatory index score and cardiovascular-kidney-metabolic syndrome: a cross-sectional study based on NHANES
Source: Front Nutr. 2025 May 9;12:1557491. doi: 10.3389/fnut.2025.1557491 (PMC12098081; doi:10.3389/fnut.2025.1557491)
Supplement: Supplementary file 5 [file Table_5.DOCX]

**Supplementary Table 5. Baseline Characteristics of Study Population Stratified by Energy-adjusted Dietary Inflammatory Index Quartiles, weighted.**

| **characteristics** | **Overall** | **Quartile 1** | **Quartile 2** | **Quartile 3** | **Quartile 4** | **p -value** |
| --- | --- | --- | --- | --- | --- | --- |
|  |  | **(-2.69, -0.06)** | **(-0.06, 0.58)** | **(0.58, 1.37)** | **(1.37, 6.96)** |  |
|  | **N=7,110** | **N=1,778** | **N=1,777** | **N=1,777** | **N=,1778** |  |
| E-DII | 0.68 ± 0.02 | -0.56 ± 0.01 | 0.25 ± 0.01 | 0.93 ± 0.01 | 2.37 ± 0.03 | <0.0001* |
| **Demographic Characteristics** | | | | | | |
| Sex, %(SE) | | | | | | |
| Male | 50.10 (0.65) | 62.38 (1.33) | 58.16 (1.53) | 47.58 (1.63) | 28.76 (1.46) | <0.0001* |
| Female | 49.90 (0.65) | 37.62 (1.33) | 41.84 (1.53) | 52.42 (1.63) | 71.24 (1.46) |  |
| Age group (years) , %(SE) | | | | | | |
| <45 | 46.39 (1.03) | 41.09 (2.01) | 47.87 (1.56) | 48.74 (1.72) | 48.38 (1.45) | 0.0027* |
| 45-64 | 37.39 (0.94) | 41.20 (1.96) | 37.46 (1.65) | 37.84 (1.68) | 32.30 (1.65) |  |
| ≥65 | 16.21 (0.63) | 17.72 (1.53) | 14.67 (1.08) | 13.43 (1.05) | 19.32 (1.24) |  |
| Race/ethnicity, %(SE) | | | | | | |
| Mexican American | 7.25 (0.66) | 7.59 (0.88) | 6.99 (0.77) | 6.73 (0.76) | 7.69 (0.87) | <0.0001* |
| Other Hispanic | 4.88 (0.46) | 5.12 (0.60) | 4.61 (0.62) | 4.54 (0.60) | 5.31 (0.65) |  |
| Non-Hispanic White | 70.95 (1.43) | 73.28 (1.71) | 72.82 (1.65) | 69.48 (1.75) | 67.59 (2.18) |  |
| Non-Hispanic Black | 9.75 (0.74) | 5.83 (0.62) | 8.82 (0.84) | 12.13 (1.01) | 12.88 (1.32) |  |
| Other Race | 7.17 (0.46) | 8.18 (0.82) | 6.76 (0.76) | 7.12 (0.72) | 6.53 (0.79) |  |
| **socioeconomic indicators** | | | | | | |
| Education level, %(SE) | | | | | | |
| ＜high school | 12.29 (0.70) | 7.61 (0.81) | 10.73 (1.00) | 14.21 (0.95) | 17.59 (1.27) | <0.0001* |
| High school | 21.10 (0.92) | 13.75 (1.20) | 19.57 (1.41) | 23.66 (1.42) | 28.82 (1.51) |  |
| ＞high school | 66.61 (1.22) | 78.63 (1.37) | 69.69 (1.70) | 62.13 (1.53) | 53.58 (1.85) |  |
| Marital status, %(SE) | | | | | | |
| Married/Living with a partner | 66.02 (0.97) | 72.55 (1.56) | 68.66 (1.52) | 63.78 (1.69) | 57.61 (1.54) | <0.0001* |
| Divorced/Separated/Widowed | 15.97 (0.66) | 12.19 (1.06) | 14.41 (0.95) | 16.20 (1.16) | 22.06 (1.36) |  |
| Never married | 18.00 (0.81) | 15.25 (1.23) | 16.93 (1.27) | 20.02 (1.32) | 20.32 (1.36) |  |
| Poverty-to-income ratio, %(SE) | | | | | | |
| <1.3 | 18.67 (0.86) | 11.62 (0.79) | 16.53 (1.18) | 20.69 (1.37) | 27.37 (1.60) | <0.0001* |
| ≥1.3, <3.5 | 35.38 (0.97) | 30.92 (1.57) | 33.89 (1.68) | 37.43 (1.81) | 40.19 (1.63) |  |
| ≥3.5 | 45.95 (1.26) | 57.46 (1.62) | 49.58 (1.90) | 41.88 (1.93) | 32.44 (1.66) |  |
| **Lifestyle Factors** | | | | | | |
| smoking status, %(SE) | | | | | | |
| Never | 56.61 (1.06) | 60.24 (1.98) | 57.32 (1.64) | 54.78 (1.89) | 53.47 (1.65) | <0.0001* |
| Former | 26.41 (0.92) | 32.00 (1.88) | 26.19 (1.50) | 23.73 (1.69) | 22.99 (1.43) |  |
| Current | 16.98 (0.67) | 7.76 (0.76) | 16.50 (1.11) | 21.49 (1.27) | 23.54 (1.40) |  |
| Alcohol consumption, %(SE) | | | | | | |
| None | 75.16 (0.82) | 67.22 (1.56) | 71.93 (1.45) | 76.86 (1.30) | 86.23 (1.13) | <0.0001* |
| Moderate intake | 8.87 (0.46) | 13.49 (1.00) | 8.68 (0.80) | 8.12 (0.90) | 4.54 (0.65) |  |
| Heavy consumption | 15.97 (0.68) | 19.29 (1.50) | 19.39 (1.33) | 15.03 (0.98) | 9.23 (0.91) |  |
| BMI (kg/m^2^), %(SE) | | | | | | |
| <25 | 31.17 (0.88) | 35.26 (1.69) | 31.96 (1.35) | 27.68 (1.36) | 29.17 (1.75) | <0.0001* |
| ≥25, <30 | 33.80 (0.70) | 36.27 (1.43) | 33.09 (1.34) | 34.04 (1.41) | 31.47 (1.49) |  |
| ≥30 | 35.04 (0.84) | 28.46 (1.60) | 34.95 (1.35) | 38.27 (1.51) | 39.36 (1.53) |  |
| Physical activity(MET-min/week) , %(SE) | | | | | | |
| <600 | 17.29 (0.56) | 12.23 (1.10) | 18.24 (1.06) | 17.53 (1.17) | 21.92 (1.30) | <0.0001* |
| ≥600 | 82.71 (0.56) | 87.77 (1.10) | 81.76 (1.06) | 82.47 (1.17) | 78.08 (1.30) |  |
| **Clinical Measurements** | | | | | | |
| WC (cm) | 98.55 ± 0.31 | 97.29 ± 0.56 | 99.09 ± 0.45 | 98.95 ± 0.47 | 98.98 ± 0.56 | 0.0306* |
| TG (mg/dL) | 118.91 ± 1.46 | 115.62 ± 2.86 | 119.92 ± 2.64 | 122.40 ± 2.73 | 117.80 ± 2.32 | 0.3853 |
| HDL-C (mg/dL) | 54.67 ± 0.30 | 55.84 ± 0.57 | 53.97 ± 0.51 | 54.56 ± 0.48 | 54.24 ± 0.58 | 0.0540 |
| FPG (mg/dL) | 104.75 ± 0.44 | 104.11 ± 0.78 | 105.14 ± 0.79 | 104.45 ± 0.74 | 105.37 ± 0.72 | 0.3278 |
| Mean SBP (mmHg) | 120.42 ± 0.30 | 119.98 ± 0.51 | 120.31 ± 0.52 | 120.17 ± 0.55 | 121.34 ± 0.52 | 0.0740 |
| Mean DBP (mmHg) | 70.02 ± 0.27 | 70.34 ± 0.42 | 70.76 ± 0.40 | 70.12 ± 0.41 | 68.68 ± 0.45 | 0.0022* |
| UACR (mg/g) | 2.37 ± 0.21 | 1.72 ± 0.20 | 2.04 ± 0.35 | 2.24 ± 0.32 | 3.68 ± 0.74 | 0.0050* |
| eGFR (mL/min/1.73 m²) | 95.72 ± 0.43 | 93.97 ± 0.75 | 96.24 ± 0.65 | 96.79 ± 0.62 | 96.02 ± 0.67 | 0.0216* |
| **Components and Outcomes** | | | | | | |
| Central obesity, %(SE) | 54.97 (1.00) | 47.68 (2.07) | 54.91 (1.42) | 56.49 (1.50) | 62.04 (1.67) | <0.0001* |
| Hypertriglyceridemia, %(SE) | 22.67 (0.73) | 20.87 (1.35) | 22.31 (1.39) | 23.70 (1.36) | 24.11 (1.24) | 0.0595 |
| Low HDL-C, %(SE) | 25.36 (0.78) | 19.93 (0.92) | 25.93 (1.36) | 24.50 (1.22) | 32.07 (1.81) | <0.0001* |
| Hypertension, %(SE) | 43.11 (1.00) | 41.62 (1.45) | 42.69 (1.66) | 42.26 (1.68) | 46.29 (1.47) | 0.0408* |
| Hyperglycemia, %(SE) | 50.26 (0.99) | 51.36 (1.75) | 51.53 (1.57) | 48.16 (1.81) | 49.74 (1.66) | 0.2559 |
| CMS, %(SE) | 35.86 (0.84) | 31.67 (1.45) | 36.61 (1.38) | 34.83 (1.45) | 41.06 (1.81) | 0.0008* |
| CKD, %(SE) | 5.58 (0.28) | 4.80 (0.58) | 4.32 (0.51) | 4.94 (0.63) | 8.71 (0.78) | <0.0001* |
| CKM, %(SE) | 3.50 (0.20) | 2.67 (0.43) | 2.98 (0.42) | 2.70 (0.46) | 5.97 (0.63) | <0.0001* |

**Note:** Data are presented as weighted means ± standard error (SE) for continuous variables and weighted percentages (SE) for categorical variables. P-values were calculated using weighted linear regression for continuous variables and weighted Chi-square test for categorical variables.

**Abbreviations:** E-DII, energy-adjusted dietary inflammatory index; BMI, body mass index; MET, metabolic equivalent of task; WC, waist circumference; TG, triglycerides; HDL-C, high-density lipoprotein cholesterol; FPG, fasting plasma glucose; SBP, systolic blood pressure; DBP, diastolic blood pressure; UACR, urinary albumin-to-creatinine ratio; eGFR, estimated glomerular filtration rate; CMS, cardiometabolic syndrome; CKD, chronic kidney disease; CKM, cardiovascular-kidney-metabolic syndrome.

*Statistically significant at P < 0.05
