# Supplementary material for: Association between dietary inflammatory index score and cardiovascular-kidney-metabolic syndrome: a cross-sectional study based on NHANES
Source: Front Nutr. 2025 May 9;12:1557491. doi: 10.3389/fnut.2025.1557491 (PMC12098081; doi:10.3389/fnut.2025.1557491)
Supplement: Supplementary file 6 [file Table_6.DOCX]

**Supplementary Table 6: Associations between E-DII and CKM syndrome and its components using multiple imputation analysis for covariates and complete case analysis**

| **E-DII** | **Multiple imputation analysis for covariates (n=10,293)** | **Complete case analysis  (n=7,110)** |
| --- | --- | --- |
| **CKM syndrome /OR (95% CI)** | | |
| Continuous | 1.24 (1.14, 1.35) *** | 1.22 (1.09, 1.37) *** |
| **Categories** | | |
| Quartile 1 | Reference | Reference |
| Quartile 2 | 1.31 (0.86, 1.98) | 1.24 (0.76, 2.04) |
| Quartile 3 | 1.31 (0.92, 1.88) | 1.13 (0.69, 1.85) |
| Quartile 4 | 2.16 (1.52, 3.08) *** | 2.07 (1.29, 3.31) ** |
| P for trend | <0.0001 | 0.0040 |
| **CKD /OR (95% CI)** | | |
| Continuous | 1.22 (1.13, 1.31) *** | 1.17 (1.07, 1.29) ** |
| **Categories** | | |
| Quartile 1 | Reference | Reference |
| Quartile 2 | 1.05 (0.76, 1.46) | 0.99 (0.66, 1.48) |
| Quartile 3 | 1.37 (1.03, 1.81) * | 1.18 (0.79, 1.79) |
| Quartile 4 | 1.90 (1.43, 2.54) *** | 1.71 (1.21, 2.42) ** |
| P for trend | <0.0001 | 0.0020 |
| **CMS syndrome /OR (95% CI)** | | |
| Continuous | 1.12 (1.07, 1.18) *** | 1.14 (1.07, 1.21) *** |
| **Categories** | | |
| Quartile 1 | Reference | Reference |
| Quartile 2 | 1.24 (1.08, 1.43) ** | 1.31 (1.13, 1.51) *** |
| Quartile 3 | 1.21 (1.03, 1.42) * | 1.21 (1.01, 1.45) * |
| Quartile 4 | 1.48 (1.25, 1.76) *** | 1.57 (1.28, 1.92) *** |
| P for trend | 0.0001 | 0.0005 |
| **Central obesity /OR (95% CI)** | | |
| Continuous | 1.10 (1.05, 1.16) *** | 1.13 (1.06, 1.20) *** |
| **Categories** | | |
| Quartile 1 | Reference | Reference |
| Quartile 2 | 1.39 (1.17, 1.66) *** | 1.37 (1.13, 1.66) ** |
| Quartile 3 | 1.34 (1.14, 1.58) *** | 1.38 (1.12, 1.69) ** |
| Quartile 4 | 1.43 (1.19, 1.72) *** | 1.48 (1.21, 1.81) *** |
| P for trend | 0.0004 | 0.0005 |
| **Hypertriglyceridemia /OR (95% CI)** | | |
| Continuous | 1.10 (1.04, 1.16) *** | 1.13 (1.06, 1.20) *** |
| **Categories** | | |
| Quartile 1 | Reference | Reference |
| Quartile 2 | 1.15 (0.96, 1.39) | 1.10 (0.88, 1.37) |
| Quartile 3 | 1.23 (1.01, 1.49) * | 1.25 (0.99, 1.57) |
| Quartile 4 | 1.29 (1.07, 1.57) * | 1.38 (1.10, 1.73) ** |
| P for trend | 0.0115 | 0.0047 |
| **Low HDL-C /OR (95% CI)** | | |
| Continuous | 1.13 (1.08, 1.18) *** | 1.13 (1.07, 1.20) *** |
| **Categories** | | |
| Quartile 1 | Reference | Reference |
| Quartile 2 | 1.22 (1.06, 1.42) ** | 1.30 (1.10, 1.53) ** |
| Quartile 3 | 1.21 (1.04, 1.40) * | 1.14 (0.97, 1.34) |
| Quartile 4 | 1.47 (1.26, 1.71) *** | 1.55 (1.27, 1.89) *** |
| P for trend | <0.0001 | 0.0005 |
| **Hypertension /OR (95% CI)** | | |
| Continuous | 1.09 (1.03, 1.14) ** | 1.09 (1.03, 1.16) ** |
| **Categories** | | |
| Quartile 1 | Reference | Reference |
| Quartile 2 | 1.09 (0.93, 1.28) | 1.14 (0.95, 1.36) |
| Quartile 3 | 1.15 (0.99, 1.33) | 1.13 (0.95, 1.35) |
| Quartile 4 | 1.31 (1.11, 1.53) ** | 1.36 (1.14, 1.64) ** |
| P for trend | 0.0021 | 0.0056 |
| **Hyperglycemia /OR (95% CI)** | | |
| Continuous | 1.07 (1.03, 1.12) ** | 1.07 (1.01, 1.13) * |
| **Categories** | | |
| Quartile 1 | Reference | Reference |
| Quartile 2 | 1.07 (0.92, 1.26) | 1.11 (0.94, 1.32) |
| Quartile 3 | 1.04 (0.87, 1.24) | 1.06 (0.87, 1.28) |
| Quartile 4 | 1.27 (1.08, 1.50) ** | 1.26 (1.03, 1.53) * |
| P for trend | 0.0132 | 0.0571 |

Model adjusted for sex, age, race, education level, marital status, poverty-to-income ratio, smoking status and physical activity measured in total METs per week.

**Abbreviations:** E-DII, energy-adjusted dietary inflammatory index; CKM, cardiovascular-kidney-metabolic syndrome; CKD, chronic kidney disease; CMS, cardiometabolic syndrome; HDL-C, high-density lipoprotein cholesterol; OR, odd ratio; CI, confidence interval; MET, metabolic equivalent of task.

*: P<0.05; **: P<0.01; ***: P<0.001
