# Supplementary material for: Association between dietary inflammatory index score and cardiovascular-kidney-metabolic syndrome: a cross-sectional study based on NHANES
Source: Front Nutr. 2025 May 9;12:1557491. doi: 10.3389/fnut.2025.1557491 (PMC12098081; doi:10.3389/fnut.2025.1557491)
Supplement: Supplementary file 7 [file Table_7.DOCX]

**Supplementary Table 7. Comparison of Characteristics Between the Final Study Population and Participants Excluded Due to Missing E-DII or CKM Data.**

| **Characteristics** | **Study population (n=7,110)** | **Missing E-DII data (n=637)** | **Standardized difference ¹** | **P-value²** | **Missing CKM data (n=746)** | **Standardized difference ¹** | **P-value²** |
| --- | --- | --- | --- | --- | --- | --- | --- |
| E-DII | 0.77 ± 1.23 | - | - | - | 1.10 ± 1.39 | 0.25 (0.18, 0.33) | <0.001 |
| **Demographic Characteristics** |  |  |  |  |  |  |  |
| Sex, n(%) |  |  | 0.13 (0.04, 0.21) | 0.003 |  | 0.19 (0.11, 0.26) | <0.001 |
| Male | 3553 (49.97%) | 358 (56.20%) |  |  | 304 (40.75%) |  |  |
| Female | 3557 (50.03%) | 279 (43.80%) |  |  | 442 (59.25%) |  |  |
| Age group (years), n(%) |  |  | 0.09 (0.01, 0.17) | 0.099 |  | 0.35 (0.27, 0.42) | <0.001 |
| <45 | 3114 (43.80%) | 307 (48.19%) |  |  | 239 (32.04%) |  |  |
| 45-64 | 2566 (36.09%) | 210 (32.97%) |  |  | 248 (33.24%) |  |  |
| ≥65 | 1430 (20.11%) | 120 (18.84%) |  |  | 259 (34.72%) |  |  |
| Race/ethnicity, n(%) |  |  | 0.30 (0.22, 0.38) | <0.001 |  | 0.16 (0.08, 0.24) | <0.001 |
| Mexican American | 984 (13.84%) | 132 (20.72%) |  |  | 102 (13.67%) |  |  |
| Other Hispanic | 684 (9.62%) | 74 (11.62%) |  |  | 65 (8.71%) |  |  |
| Non-Hispanic White | 3287 (46.23%) | 211 (33.12%) |  |  | 309 (41.42%) |  |  |
| Non-Hispanic Black | 1316 (18.51%) | 117 (18.37%) |  |  | 186 (24.93%) |  |  |
| Other Race | 839 (11.80%) | 103 (16.17%) |  |  | 84 (11.26%) |  |  |
| **Socioeconomic Indicators** |  |  |  |  |  |  |  |
| Education level, n(%) |  |  | 0.34 (0.26, 0.43) | <0.001 |  | 0.27 (0.19, 0.34) | <0.001 |
| <High school | 1313 (18.47%) | 205 (32.18%) |  |  | 211 (28.48%) |  |  |
| High school | 1537 (21.62%) | 143 (22.45%) |  |  | 174 (23.48%) |  |  |
| >High school | 4260 (59.92%) | 289 (45.37%) |  |  | 356 (48.04%) |  |  |
| Marital status, n(%) |  |  | 0.09 (0.01, 0.17) | 0.080 |  | 0.28 (0.20, 0.35) | <0.001 |
| Married/Living with a partner | 4430 (62.31%) | 369 (57.93%) |  |  | 384 (51.47%) |  |  |
| Divorced/Separated/Widowed | 1365 (19.20%) | 141 (22.14%) |  |  | 231 (30.97%) |  |  |
| Never married | 1315 (18.50%) | 127 (19.94%) |  |  | 131 (17.56%) |  |  |
| Poverty-to-income ratio, n(%) |  |  | 0.25 (0.17, 0.34) | <0.001 |  | 0.29 (0.21, 0.37) | <0.001 |
| <1.3 | 1999 (28.12%) | 221 (38.43%) |  |  | 261 (38.72%) |  |  |
| ≥1.3, <3.5 | 2660 (37.41%) | 211 (36.70%) |  |  | 261 (38.72%) |  |  |
| ≥3.5 | 2451 (34.47%) | 143 (24.87%) |  |  | 152 (22.55%) |  |  |
| **Lifestyle Factors** |  |  |  |  |  |  |  |
| Smoking status, n(%) |  |  | 0.21 (0.12, 0.29) | <0.001 |  | 0.01 (-0.07, 0.08) | 0.976 |
| Never | 4028 (56.65%) | 339 (53.30%) |  |  | 421 (56.43%) |  |  |
| Former | 1804 (25.37%) | 131 (20.60%) |  |  | 192 (25.74%) |  |  |
| Current | 1278 (17.97%) | 166 (26.10%) |  |  | 133 (17.83%) |  |  |
| Alcohol consumption, n(%) |  |  | 0.15 (0.07, 0.23) | <0.001 |  | 0.17 (0.09, 0.25) | <0.001 |
| None | 5609 (78.89%) | 474 (74.41%) |  |  | 636 (85.25%) |  |  |
| Moderate intake | 566 (7.96%) | 45 (7.06%) |  |  | 46 (6.17%) |  |  |
| Heavy consumption | 935 (13.15%) | 118 (18.52%) |  |  | 64 (8.58%) |  |  |
| BMI (kg/m²), n(%) |  |  | 0.10 (0.02, 0.19) | 0.038 |  | 0.15 (0.07, 0.23) | <0.001 |
| <25 | 2111 (29.72%) | 218 (34.44%) |  |  | 187 (28.08%) |  |  |
| ≥25, <30 | 2418 (34.05%) | 208 (32.86%) |  |  | 191 (28.68%) |  |  |
| ≥30 | 2573 (36.23%) | 207 (32.70%) |  |  | 288 (43.24%) |  |  |
| Physical activity (MET-min/week), n(%) |  |  | 0.05 (-0.04, 0.14) | 0.281 |  | 0.22 (0.12, 0.31) | <0.001 |
| <600 | 1302 (18.31%) | 79 (16.36%) |  |  | 119 (27.29%) |  |  |
| ≥600 | 5808 (81.69%) | 404 (83.64%) |  |  | 317 (72.71%) |  |  |
| **Clinical Measurements** |  |  |  |  |  |  |  |
| Waist circumference (cm) | 98.68 ± 16.03 | 97.59 ± 16.13 | 0.07 (-0.01, 0.15) | 0.099 | 99.81 ± 17.71 | 0.07 (-0.03, 0.16) | 0.158 |
| Triglycerides (mg/dL) | 120.56 ± 95.59 | 125.41 ± 100.51 | 0.05 (-0.03, 0.13) | 0.222 | 130.31 ± 151.51 | 0.08 (-0.00, 0.16) | 0.020 |
| HDL-C (mg/dL) | 54.11 ± 15.70 | 52.64 ± 15.91 | 0.09 (0.01, 0.17) | 0.023 | 53.72 ± 16.04 | 0.02 (-0.06, 0.11) | 0.543 |
| Fasting plasma glucose (mg/dL) | 107.45 ± 31.88 | 111.71 ± 39.18 | 0.12 (0.04, 0.20) | 0.002 | 113.46 ± 42.56 | 0.16 (0.08, 0.24) | <0.001 |
| Systolic blood pressure (mmHg) | 122.15 ± 17.62 | 124.82 ± 19.92 | 0.14 (0.06, 0.22) | <0.001 | 128.44 ± 22.17 | 0.31 (0.22, 0.41) | <0.001 |
| Diastolic blood pressure (mmHg) | 69.61 ± 12.70 | 70.65 ± 13.66 | 0.08 (-0.00, 0.16) | 0.050 | 68.35 ± 14.72 | 0.09 (-0.00, 0.19) | 0.039 |
| UACR (mg/g) | 3.30 ± 24.40 | 7.48 ± 53.63 | 0.10 (0.02, 0.18) | <0.001 | 12.29 ± 72.74 | 0.17 (0.08, 0.25) | <0.001 |
| eGFR (mL/min/1.73m²) | 95.90 ± 22.01 | 98.37 ± 22.61 | 0.11 (0.03, 0.19) | 0.007 | 86.60 ± 29.40 | 0.36 (0.27, 0.44) | <0.001 |
| **Components and Outcomes** |  |  |  |  |  |  |  |
| Central obesity, n(%) |  |  | 0.10 (0.02, 0.18) | 0.015 |  | 0.06 (-0.04, 0.16) | 0.223 |
| No | 3204 (45.06%) | 319 (50.08%) |  |  | 183 (42.07%) |  |  |
| Yes | 3906 (54.94%) | 318 (49.92%) |  |  | 252 (57.93%) |  |  |
| Hypertriglyceridemia, n(%) |  |  | 0.01 (-0.08, 0.09) | 0.884 |  | 0.04 (-0.04, 0.12) | 0.353 |
| No | 5465 (76.86%) | 488 (76.61%) |  |  | 477 (75.24%) |  |  |
| Yes | 1645 (23.14%) | 149 (23.39%) |  |  | 157 (24.76%) |  |  |
| Low HDL-C, n(%) |  |  | 0.05 (-0.03, 0.13) | 0.242 |  | 0.07 (-0.01, 0.15) | 0.075 |
| No | 5198 (73.11%) | 452 (70.96%) |  |  | 447 (69.84%) |  |  |
| Yes | 1912 (26.89%) | 185 (29.04%) |  |  | 193 (30.16%) |  |  |
| Hypertension, n(%) |  |  | 0.02 (-0.07, 0.10) | 0.707 |  | 0.61 (0.52, 0.70) | <0.001 |
| No | 3751 (52.76%) | 341 (53.53%) |  |  | 140 (24.35%) |  |  |
| Yes | 3359 (47.24%) | 296 (46.47%) |  |  | 435 (75.65%) |  |  |
| Hyperglycemia, n(%) |  |  | 0.13 (0.05, 0.21) | 0.002 |  | 0.11 (0.04, 0.19) | 0.003 |
| No | 3331 (46.85%) | 257 (40.35%) |  |  | 307 (41.15%) |  |  |
| Yes | 3779 (53.15%) | 380 (59.65%) |  |  | 439 (58.85%) |  |  |
| CMS, n(%) |  |  | 0.00 (-0.08, 0.08) | 0.986 |  | 0.36 (0.21, 0.51) | <0.001 |
| No | 4384 (61.66%) | 393 (61.70%) |  |  | 79 (44.13%) |  |  |
| Yes | 2726 (38.34%) | 244 (38.30%) |  |  | 100 (55.87%) |  |  |
| CKD, n(%) |  |  | 0.04 (-0.04, 0.12) | 0.352 |  | 0.45 (0.36, 0.54) | <0.001 |
| No | 6611 (92.98%) | 586 (91.99%) |  |  | 416 (77.47%) |  |  |
| Yes | 499 (7.02%) | 51 (8.01%) |  |  | 121 (22.53%) |  |  |
| CKM, n(%) |  |  | 0.03 (-0.05, 0.11) | 0.466 |  | 0.02 (-0.07, 0.11) | 0.634 |
| No | 6797 (95.60%) | 605 (94.98%) |  |  | 470 (95.14%) |  |  |
| Yes | 313 (4.40%) | 32 (5.02%) |  |  | 24 (4.86%) |  |  |

**Note:** Data are presented as mean ± standard deviation for continuous variables and number (percentage) for categorical variables. Standardized differences are presented with 95% confidence intervals in parentheses.

**Abbreviations:** E-DII, energy-adjusted dietary inflammatory index; BMI, body mass index; MET, metabolic equivalent of task; WC, waist circumference; TG, triglycerides; HDL-C, high-density lipoprotein cholesterol; FPG, fasting plasma glucose; SBP, systolic blood pressure; DBP, diastolic blood pressure; UACR, urinary albumin-to-creatinine ratio; eGFR, estimated glomerular filtration rate; CMS, cardiometabolic syndrome; CKD, chronic kidney disease; CKM, cardiovascular-kidney-metabolic syndrome.
