# Supplementary material for: Association between dietary inflammatory index score and cardiovascular-kidney-metabolic syndrome: a cross-sectional study based on NHANES
Source: Front Nutr. 2025 May 9;12:1557491. doi: 10.3389/fnut.2025.1557491 (PMC12098081; doi:10.3389/fnut.2025.1557491)
Supplement: Supplementary file 8 [file Table_8.DOCX]

**Supplementary Table 8. E-value Analysis: Assessment of Potential Impact of Unmeasured Confounding on Associations Between E-DII and CKM Syndrome and Its Components in Model 3.**

| **E-DII** | **OR (95% CI)** | **E-value** | **E-value for CI limit** |
| --- | --- | --- | --- |
| **CKM Syndrome** |  |  |  |
| Continuous | 1.22 (1.09, 1.37) | 1.44 | 1.26 |
| Q4 vs Q1 | 2.07 (1.29, 3.31) | 2.23 | 1.53 |
| **CKD** |  |  |  |
| Continuous | 1.17 (1.07, 1.29) | 1.38 | 1.22 |
| Q4 vs Q1 | 1.71 (1.21, 2.42) | 1.94 | 1.43 |
| **CMS** |  |  |  |
| Continuous | 1.14 (1.07, 1.21) | 1.34 | 1.22 |
| Q4 vs Q1 | 1.57 (1.28, 1.92) | 1.82 | 1.52 |
| **CMS Components (Q4 vs Q1)** |  |  |  |
| Central obesity | 1.48 (1.21, 1.81) | 1.73 | 1.43 |
| Hypertriglyceridemia | 1.38 (1.10, 1.73) | 1.63 | 1.28 |
| Low HDL-C | 1.55 (1.27, 1.89) | 1.80 | 1.51 |
| Hypertension | 1.36 (1.14, 1.64) | 1.61 | 1.34 |
| Hyperglycemia | 1.26 (1.03, 1.53) | 1.49 | 1.14 |

**Notes:**

1. Model 3 was adjusted for sex, age, race, education level, marital status, poverty-to-income ratio, smoking status and physical activity measured in total METs per week.
2. E-value represents the minimum strength of association that an unmeasured confounder would need to have with both the exposure (E-DII) and the outcome (CKM syndrome and its components) to fully explain away the observed association.
3. E-value for CI limit represents the minimum strength of association that an unmeasured confounder would need to have to shift the confidence interval to include the null value.
4. Higher E-values indicate that stronger unmeasured confounding would be needed to explain away the observed associations.
